# Sedentary lifestyle, physical activity, and gastrointestinal diseases: evidence from mendelian randomization analysis

**DOI:** 10.1016/j.ebiom.2024.105110

**Published:** 2024-04-06

**Authors:** Jie Chen, Xixian Ruan, Tian Fu, Shiyuan Lu, Dipender Gill, Zixuan He, Stephen Burgess, Edward L. Giovannucci, Susanna C. Larsson, Minzi Deng, Shuai Yuan, Xue Li

**Affiliations:** a School of Public Health and the Second Affiliated Hospital, Zhejiang University School of Medicine, Hangzhou, Zhejiang, China; b Department of Gastroenterology, The Third Xiangya Hospital, Central South University, Changsha, China; c Department of Gastroenterology, The Second Affiliated Hospital of Zhejiang University School of Medicine, Hangzhou, China; d Department of Epidemiology and Biostatistics, School of Public Health, Imperial College London, London, UK; e Department of Gastroenterology, Changhai Hospital, Second Military Medical University/Naval Medical University, Shanghai, China; f MRC Biostatistics Unit, University of Cambridge, Cambridge, UK; g Department of Public Health and Primary Care, University of Cambridge, Cambridge, UK; h Department of Epidemiology, Harvard T H Chan School of Public Health, Boston, MA, USA; i Department of Nutrition, Harvard T H Chan School of Public Health, Boston, MA, USA; j Unit of Cardiovascular and Nutritional Epidemiology, Institute of Environmental Medicine, Karolinska Institutet, Stockholm, Sweden; k Unit of Medical Epidemiology, Department of Surgical Sciences, Uppsala University, Uppsala, Sweden; l Centre for Global Health Research, Usher Institute, University of Edinburgh, Edinburgh, UK

**Keywords:** Gastrointestinal diseases, Leisure screen time, Mendelian randomization, Physical activity, Sedentary lifestyle

## Abstract

**Background:**

The causal associations of physical activity and sedentary behavior with the risk of gastrointestinal disease are unclear. We performed a Mendelian randomization analysis to examine these associations.

**Methods:**

Genetic instruments associated with leisure screen time (LST, an indicator of a sedentary lifestyle) and moderate-to-vigorous intensity physical activity (MVPA) at the genome-wide significance (*P* < 5 × 10^−8^) level were selected from a genome-wide association study. Summary statistics for gastrointestinal diseases were obtained from the UK Biobank study, the FinnGen study, and large consortia. Multivariable MR analyses were conducted for genetically determined LST with adjustment for MVPA and vice versa. We also performed multivariable MR with adjustment for genetically proxied smoking, body mass index (BMI), waist-to-hip ratio, type 2 diabetes, and fasting insulin for both exposures.

**Findings:**

Genetically proxied longer LST was associated with an increased risk of gastrointestinal reflux, gastric ulcer, duodenal ulcer, chronic gastritis, irritable bowel syndrome, diverticular disease, Crohn’s disease, ulcerative colitis, non-alcoholic fatty liver disease, alcoholic liver disease, cholangitis, cholecystitis, cholelithiasis, acute pancreatitis, chronic pancreatitis, and acute appendicitis. Most associations remained after adjustment for genetic liability to MVPA. Genetic liability to MVPA was associated with decreased risk of gastroesophageal reflux, gastric ulcer, chronic gastritis, irritable bowel syndrome, cholecystitis, cholelithiasis, acute and chronic pancreatitis. The associations attenuated albeit directionally remained after adjusting for genetically predicted LST. Multivariable MR analysis found that BMI and type 2 diabetes mediated the associations of LST and MVPA with several gastrointestinal diseases.

**Interpretation:**

The study suggests that a sedentary lifestyle may play a causal role in the development of many gastrointestinal diseases.

**Funding:**

Natural Science Fund for Distinguished Young Scholars of Zhejiang Province (LR22H260001), Natural Science Foundation of Hunan Province (2021JJ30999), 10.13039/501100003793Swedish Heart-Lung Foundation (Hjärt-Lungfonden, 20210351), 10.13039/501100004359Swedish Research Council (Vetenskapsrådet, 2019-00977), 10.13039/501100002794Swedish Cancer Society (10.13039/501100002794Cancerfonden), the 10.13039/100010269Wellcome Trust (225790/7/22/Z), United Kingdom Research and Innovation Medical Research Council (MC_UU_00002/7) and 10.13039/501100018956National Institute for Health Research Cambridge Biomedical Research Centre (NHIR203312).

**Research in context**

**Evidence before this study**

Although evidence from observational studies has suggested that physical activity was associated with risk of several gastrointestinal diseases, whether these associations were causal remains uncertain. Besides, the relationship between sedentary behavior and gastrointestinal diseases has been scarcely investigated.

**Added value of this study**

Genetically predicted longer leisure screen time (LST) was associated with an increased risk of 16 gastrointestinal diseases, whereas genetic liability to moderate-to-vigorous intensity physical activity (MVPA) was inversely associated with 8 gastrointestinal diseases. Genetically proxied body mass index, fasting insulin levels, and type 2 diabetes partially mediated the associations of LST and MVPA with several gastrointestinal diseases.

**Implications of all the available evidence**

Public health efforts to promote increased physical activity and decreased sedentary time can be an effective strategy to prevent gastrointestinal diseases.

## Introduction

Insufficient physical activity affects approximately 27.5% of the global population, with a higher prevalence of 42.3% in high-income countries, significantly contributing to the burden of disease.^1^ Previous population-based observational studies found that high physical activity levels were associated with a low risk of many gastrointestinal diseases, including gastroesophageal reflux disease,^2^ esophageal cancer,^3^ colorectal cancer,^4^ cholecystitis,^5^ cholelithiasis^6^ and non-alcoholic fatty liver disease.^7^ The causal potential of the association for liver disease was further supported by a meta-analysis of 11 randomized controlled trials which found that exercise improved hepatic steatosis and serum alanine aminotransferase levels in patients with non-alcoholic fatty liver disease.^8^ However, the causality of most of these associations is not fully established due to potential limitations of observational studies, like residual confounding, misclassification, and reverse causation.^9^ Sedentary behaviors may also influence human health and the risk caused by a sedentary lifestyle cannot be offset by physical activity.^10^^,^^11^ Nevertheless, few studies have been conducted to explore the associations between sedentary behavior and the risk of gastrointestinal diseases; the causal associations of sedentary behavior and physical activity with the risk of developing gastrointestinal diseases remain to be elucidated.

Mendelian randomization (MR) is an epidemiological approach that utilizes genetic variants as instrumental variables to strengthen causal inference.^12^ Genetic variants are randomly assorted at conception and thus are not subject to confounding. Specifically, our investigation employed the multivariable Mendelian randomization (MVMR) technique, which enables us to mitigate potential pleiotropy or explore mediation effects by estimating associations conditionally on potential pleiotropic traits or mediators, respectively. Regarding mediation estimation, this investigation allows us to discern the underlying mechanisms, shedding light on the specific processes by which these lifestyle factors impact the risk and development of gastrointestinal diseases. Herein, we conducted an outcome-wide MR investigation to unravel the intricate associations between sedentary behavior, physical activity, and a wide spectrum of gastrointestinal diseases.

## Methods

**Fig. 1:**
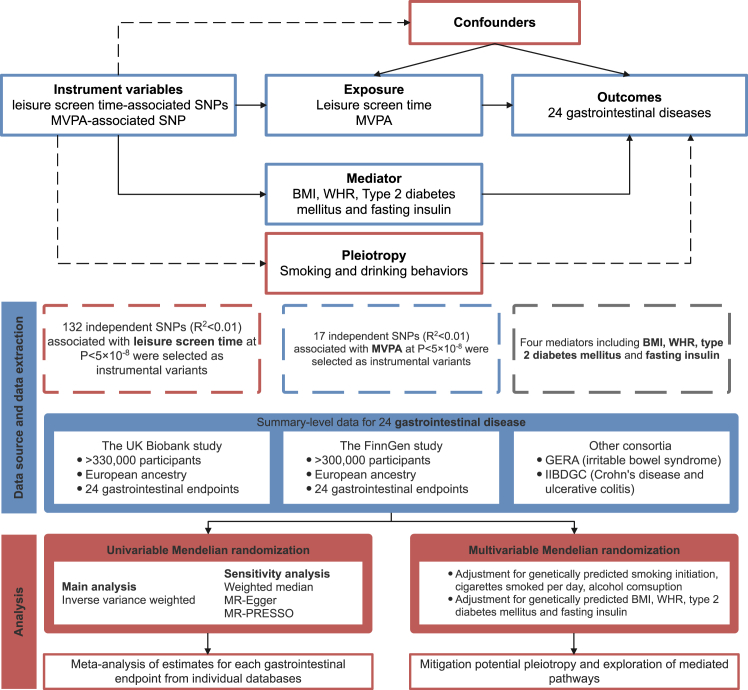
**Study design.** MVPA, moderate-to-vigorous intensity physical activity during leisure time; MR, Mendelian randomization; GERA Resource for Genetic Epidemiology Research on Aging; IIBDGC, the International Inflammatory Bowel Disease Genetics Consortium; MR-PRESSO, Mendelian randomization pleiotropy residual sum, and outlier; BMI, body mass index; WHR, waist to hip ratio.

We used leisure screen time (LST) as an indicator of sedentary behavior and moderate-to-vigorous intensity physical activity during leisure time (MVPA) for physical activity. The study design is presented in Fig. 1. The study was based on data from large genome-wide association studies (GWASs) including the UK Biobank study,^13^ the FinnGen study,^14^ the Resource for Genetic Epidemiology Research on Aging (GERA),^15^ and the International Inflammatory Bowel Disease Genetics Consortium (IIBDGC).^16^ Detailed information on used data sources is shown in Supplementary Table S1. MR estimates were generated separately in each outcome dataset and then combined for each gastrointestinal endpoint using meta-analysis. We first employed the univariable MR analysis to investigate the associations of genetically predicted LST and MVPA with the risk of 24 gastrointestinal diseases. MR has three key assumptions: 1) genetic variants used as the instrumental variable are strongly linked to the exposure; 2) genetic instruments are not associated with confounder, and 3) genetic instruments affect the outcome only through exposure. To minimize potential pleiotropy from smoking and alcohol consumption, MVMR was applied to the identified relationships. Furthermore, mediation effects of body mass index (BMI), waist-to-hip ratio (WHR), type 2 diabetes, and fasting insulin in the LST-outcome or MVPA-outcome association were examined.

### Instrumental variable selection

Genetic variants for LST (N = 606,820) and MVPA (N = 526,725) were extracted from a GWAS meta-analysis of 51 studies of European populations.^17^ The two phenotypes were based on self-reported data from the questionnaires.^17^ In the original GWAS article, genetic associations for LST and MVPA were derived using additive genetic models. The analysis accounted for family relatedness (where appropriate) and adjusted for age, age-squared, principal components reflecting population structure, and additional study-specific covariates. Single nucleotide polymorphisms (SNPs) at the genome-wide significance threshold (*P* < 5 × 10^−8^) were identified. These genetic variants were clumped for linkage disequilibrium (defined as r^2^ > 0.01), leaving 132 SNPs as instrumental variables for LST and 17 SNPs for MVPA. Detailed information on SNPs used is shown in Supplementary Table S2. We performed a sensitivity analysis using the genetic instruments for LST and MVPA selected under a more stringent linkage disequilibrium threshold of r^2^ = 0.001. There is a sample overlap of approximately 67% between the exposure dataset and one of the outcome datasets (i.e., the UK Biobank). However, there is no overlap between the exposure dataset and the other outcome datasets including the FinnGen study, IIBDGC, and GERA. We examined the PhenoScanner V2 platform^18^ to identify the genetic variants strongly associated with gastrointestinal diseases and then performed the MR analysis excluding these identified instruments.

### Gastrointestinal disease data sources

Genetic associations with 24 gastrointestinal diseases were obtained from the UK Biobank study,^19^ the FinnGen study,^14^ and two large consortia, including IIBDGC^16^ and GERA.^15^ The UK Biobank study is a large multicenter cohort study initiated in the United Kingdom between 2006 and 2010 and comprises approximately half a million individuals.^19^ Summary-level statistics on gastrointestinal outcomes were derived from GWAS summary statistics of European participants from Lee lab (https://www.leelabsg.org/resources) wherein endpoints were defined using codes of the International Classification of Diseases 9th Revision (ICD-9) and ICD-10. Genetic associations were adjusted for sex, birth year, and the first four genetic principal components. We also obtained data on the associations between exposure-SNPs and gastrointestinal diseases from the latest FinnGen study R9 release.^14^ The FinnGen study combines genetic data from nationwide biobanks and disease status from structured national healthcare databases. The gastrointestinal diseases were defined by the ICD codes (ICD-8, ICD-9, and ICD-10). Genetic associations were adjusted for sex, age, genotyping batch, and 10 principal components. ICD codes used to define the outcomes in UK Biobank and FinnGen are presented in Supplementary Table S3. Additionally, summary-level data from the IIBDGC were extracted for Crohn’s disease (5956 cases and 14,927 controls) and ulcerative colitis (6968 cases and 20,464 controls)^16^ and summary-level data from the GERA were extracted for irritable bowel syndrome (3117 cases and 53,520 controls).^15^

### Data sources for smoking, alcohol consumption, body mass index, diabetic traits

The instrumental variables for smoking initiation, cigarettes smoked per day, alcohol consumption, BMI, WHR, type 2 diabetes mellitus liability, and fasting insulin were extracted from the corresponding latest publicly available GWASs, respectively.^20^, ^21^, ^22^, ^23^ Summary-level data on smoking initiation from GWAS and Sequencing Consortium of Alcohol and Nicotine use consortium (GSCAN) were extracted where the phenotype reflected smoking status.^20^ Genetic associations with cigarettes smoked per day were obtained from GSCAN which capture the heaviness of smoking.^20^ Genetic associations with alcohol consumption were also derived from GSCAN including 941,280 European-ancestry individuals.^20^ Genetic associations with BMI and WHR were obtained from the largest GWAS which contained up to 694,649 individuals of European ancestry.^21^ Genetic associations with fasting insulin were obtained from a GWAS with 151,013 European-ancestry individuals.^22^ Summary-level data on type 2 diabetes liability were selected from a meta-analysis GWAS which including 148,726 cases and 965,732 controls.^23^ Detailed information on these studies is shown in Supplementary Table S1.

### Statistical analysis

The primary MR estimates were calculated using the inverse-variance weighted (IVW) method under multiplicative random effects. The IVW method provides the most precise estimate under the assumption that all instruments are valid. Estimates for each outcome from different sources were combined using the fixed-effects meta-analysis method. Cochran’s Q value was employed to evaluate the heterogeneity among SNPs’ estimates. Three sensitivity analyses were conducted including the weighted median,^24^ MR-Egger,^25^ and Mendelian randomization pleiotropy residual sum and outlier (MR-PRESSO)^26^ to examine the robustness of the associations and to detect potential horizontal pleiotropy. The weighted median method provides a consistent estimate if >50% of the weight comes from valid instrumental variables.^24^ Although MR-Egger regression provides an estimate usually underpowered, the embedded intercept test can detect potential horizontal pleiotropy.^25^ MR-PRESSO can identify horizontal pleiotropic outliers and provide a corrected estimate using the fixed-effects IVW after the removal of the outliers.^26^ The MR Steiger test of directionality was performed to examine direction of identified association between exposure and outcome. Leave-one-out sensitivity analysis was conducted to rule out the possibility that the observed association between physical activity instrumented by 14 SNPs and gastrointestinal disease was driven by a single genetic variant.

Given a strong genetic correlation between LST and MVPA (rho = −0.49), we performed multivariable MR (MVMR) analyses with mutual adjustment to explore the independent effects of LST and MVPA on gastrointestinal diseases. To minimize pleiotropy from smoking and alcohol consumption, we performed an MVMR analysis on the associations between LST or MVPA and the risk of gastrointestinal diseases with adjustment for genetic predisposition to smoking (including smoking initiation and cigarettes smoked per day) and alcohol consumption. In brief, both the exposure and the adjusted factor were treated as the independent variables in the model. Thus, we used selected genetic variants associated with LST (or MVPA) and smoking initiation (or cigarettes smoked per day or alcohol consumption) as the instrumental variables in this MVMR analysis. We obtained estimates for both the exposure and adjusted factor in relation to gastrointestinal diseases after mutual adjustment. Given LST and MVPA as the exposures’ interests, we only reported their adjusted estimates.

Likewise, MVMR analyses on the associations between LST or MVPA and the risk of gastrointestinal diseases were also conducted with adjustments for genetically predicted BMI, WHR, type 2 diabetes, and fasting insulin, respectively. Leveraging the MVMR results, the mediation of these factors in the associations between LST, MVPA, and the risk of gastrointestinal disease was explored. When both the total effect and the mediated effect align in the same direction, it indicates the presence of a mediating effect. The mediation effect was calculated by the formula: 1–direct effect (the estimate after adjusting for the mediator)/total effect (the estimate in the univariable MR analysis).

Power estimation was performed for each gastrointestinal outcome. *F*-statistic was calculated for each SNP and an *F*-statistic > 10 indicated a reliable instrument variable. The conditional *F*-statistic was calculated to characterize instrument strengths in MVMR (Supplementary Table S4). However, the power analysis and variance explained were not available for MVPA due to its binary phenotype. Odds ratios (ORs) and confidence intervals (CIs) of gastrointestinal diseases were scaled to a one-standard-deviation (SD) increase in hours/day of genetically predicted LST and a one-unit increase in log odds of genetic liability to MVPA.^17^ As the standard deviation (SD) of the genetically predicted LST was not reported in the original GWAS, we performed a weighted analysis to derive an approximate SD using the subset of cohorts that provided mean and SD data. Each cohort was weighted by its respective sample size. The weighted SD of overall sample was approximately 1.9 h/day. The Benjamini-Hochberg method that controls the false discovery rate (FDR) was applied to correct for multiple testing. The association with a Benjamini–Hochberg adjusted *P* value < 0.05 was deemed statistically significant. All analyses were two-sided and performed using the TwoSampleMR,^27^ MendelianRandomization,^24^ and MRPRESSO^26^ R packages in R software 4.2.2.

### Ethics

All included GWASs had been approved by corresponding institutional review boards and ethical committees. All participants had signed informed consent.

### Role of funders

The funders did not participate in the study’s design and implementation, data collection, management, analysis, or interpretation. They were also not involved in the preparation, review, or approval of the manuscript, nor in the decision to submit it for publication.

## Results

There was no indication of weak instruments for LST (*F*-statistic for each genetic variant was above 10) (Supplementary Table S2). However, not all the conditional F-statistics of instruments were more than 10 in MVMR (Supplementary Table S4). Power calculations showed that there was 80% power to detect the OR ranging from 1.13 to 1.94 for each outcome (Supplementary Table S5). Using PhenoScanner, we identified rs13107325 associated with Crohn’s disease (*P* = 2.40 × 10^−8^), and rs7615206 associated with ulcerative colitis (*P* = 6.90 × 10^−10^) (Supplementary Table S6). Neither of the two SNPs was included in the MR analysis for Crohn’s disease or ulcerative colitis.

### LST and gastrointestinal disease

**Fig. 2:**
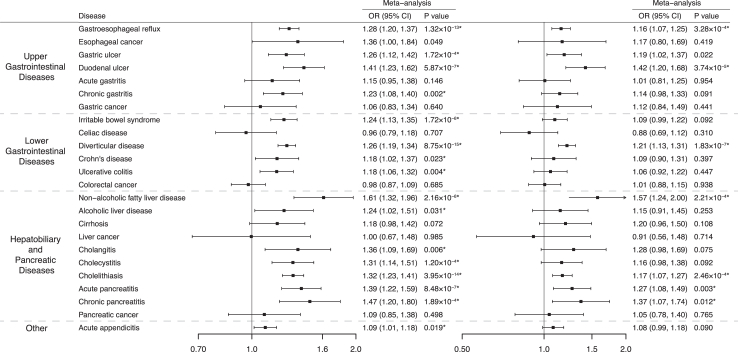
**Associations of genetically predicted leisure screen time with 24 gastrointestinal diseases in univariable Mendelian randomization and multivariable Mendelian randomization (adjustment for moderate-to-vigorous intensity physical activity).** ∗ Significant association after multiple testing. The estimate of irritable bowel syndrome was meta-analyzed by combining estimates from the UK Biobank study, the FinnGen study, and the Genetic Epidemiology Research on Aging consortium; the estimates of Crohn’s disease and ulcerative colitis were meta-analyzed by combining estimates from the UK Biobank study, the FinnGen study and the International Inflammatory Bowel Disease Genetics Consortium; the estimates of other gastrointestinal disease were meta-analysis by combining estimates from the UK Biobank study and the FinnGen study. ORs for gastrointestinal diseases were scaled to genetically predicted one standard deviation increase in hours/day of leisure screen time.

After multiple comparison corrections, genetically predicted longer LST (per SD) was associated with an increased risk of 16 of 24 gastrointestinal diseases including gastrointestinal reflux (OR 1.28, 95% CI 1.20–1.37; *P* = 1.32 × 10^−13^), gastric ulcer (OR 1.26, 95% CI 1.12–1.42; *P* = 1.72 × 10^−4^), duodenal ulcer (OR 1.41, 95% CI 1.23–1.62; *P* = 5.87 × 10^−7^), chronic gastritis (OR 1.23, 95% CI 1.08–1.40; *P* = 0.002), irritable bowel syndrome (OR 1.24, 95% CI 1.13–1.35; *P* = 1.72 × 10^−6^), diverticular disease (OR 1.26, 95% CI 1.19–1.34; *P* = 8.75 × 10^−15^), Crohn’s disease (OR 1.18, 95% CI 1.02–1.37; *P* = 0.023), ulcerative colitis (OR 1.18, 95% CI 1.06–1.32; *P* = 0.004), non-alcoholic fatty liver disease (OR 1.61, 95% CI 1.32–1.96; *P* = 2.16 × 10^−6^), alcoholic liver disease (OR 1.24, 95% CI 1.02–1.51; *P* = 0.031), cholangitis (OR 1.36, 95% CI 1.09–1.69; *P* = 0.006), cholecystitis (OR 1.31, 95% CI 1.14–1.51; *P* = 1.20 × 10^−4^), cholelithiasis (OR 1.32, 95% CI 1.23–1.41; *P* = 3.95 × 10^−14^), acute pancreatitis (OR 1.39, 95% CI 1.22–1.59; *P* = 8.48 × 10^−7^), chronic pancreatitis (OR 1.47, 95% CI 1.20–1.80; *P* = 1.89 × 10^−4^), and acute appendicitis (OR 1.09, 95% CI 1.01–1.18; *P* = 0.019) (Fig. 2 and Supplementary Table S7). Heterogeneity in SNP estimates was detected in the analysis for gastroesophageal reflux, chronic gastritis, diverticular disease, non-alcoholic fatty liver disease, cholelithiasis, chronic pancreatitis, and acute appendicitis in FinnGen. Most associations were directionally consistent in sensitivity analyses (Supplementary Table S8). The MR-Egger intercept test detected potential horizontal pleiotropy in the analysis for gastroesophageal reflux in FinnGen (*P* for intercept < 0.05). However, this association persisted in MR-PRESSO analysis after the removal of an outlier (Supplementary Table S8). MR-PRESSO also detected 1–2 outliers in the analyses for gastric ulcer, Crohn’s disease, ulcerative colitis, and cholelithiasis; however, these associations remained after removing the outliers (Supplementary Table S8). The direction of identified associations was confirmed by the MR Steiger test (Supplementary Table S9).

The associations remained in the sensitivity analysis using a stringent threshold of linkage (Supplementary Table S10). In MVMR adjustment for genetic liability to MVPA, genetically predicted LST was associated with gastroesophageal reflux, gastric ulcer, duodenal ulcer, diverticular disease, non-alcoholic fatty liver disease, cholelithiasis, acute pancreatitis and chronic pancreatitis (*P* < 0.05) although slightly attenuated (Fig. 2 and Supplementary Table S11).

### MVPA and gastrointestinal diseases

**Fig. 3:**
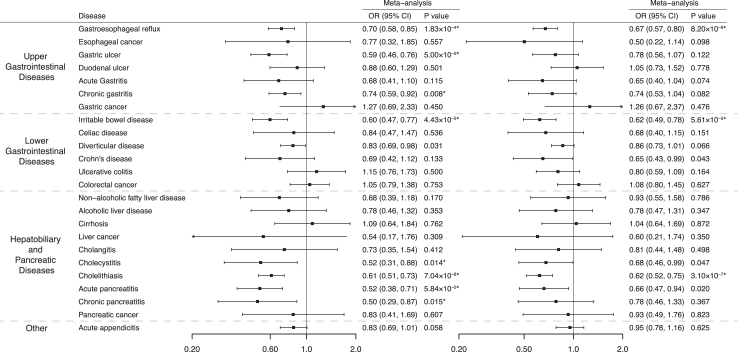
**Associations of genetically predicted moderate-to-vigorous intensity physical activity with 24 gastrointestinal diseases in univariable Mendelian randomization and multivariable Mendelian randomization (adjustment for leisure screen time).** ∗ Significant association after multiple testing. The estimate of irritable bowel syndrome was meta-analyzed by combining estimates from the UK Biobank study, the FinnGen study, and the Genetic Epidemiology Research on Aging consortium; the estimates of Crohn’s disease and ulcerative colitis were meta-analyzed by combining estimates from the UK Biobank study, the FinnGen study and the International Inflammatory Bowel Disease Genetics Consortium; the estimates of other gastrointestinal disease were meta-analysis by combining estimates from the UK Biobank study and the FinnGen study. ORs for gastrointestinal diseases were scaled to genetically predicted one unit increase in log odds of moderate-to-vigorous intensity physical activity.

Genetic predisposition to MVPA (being active vs inactive) was associated with a decreased risk of 8 gastrointestinal diseases including gastroesophageal reflux (OR 0.70, 95% CI 0.58–0.85; *P* = 1.83 × 10^−4^), gastric ulcer (OR 0.59, 95% CI 0.46–0.76; *P* = 5.00 × 10^−5^), chronic gastritis (OR 0.74, 95% CI 0.59–0.92; *P* = 0.008), irritable bowel syndrome (OR 0.60, 95% CI 0.47–0.77; *P* = 4.43 × 10^−5^), cholecystitis (OR 0.52, 95% CI 0.31–0.88; *P* = 0.014), cholelithiasis (OR 0.61, 95% CI 0.51–0.73; *P* = 7.04 × 10^−8^), acute pancreatitis (OR 0.52, 95% CI 0.38–0.71; *P* = 5.84 × 10^−5^), and chronic pancreatitis (OR 0.50, 95% CI 0.29–0.87; *P* = 0.015) after correcting for multiple testing (Fig. 3 and Supplementary Table S7). There is heterogeneity in SNP estimates for the analysis of gastroesophageal reflux in the UK Biobank (Supplementary Table S13). Estimates across sensitivity analyses were directionally consistent. The MR-Egger intercept test did not detect any indication of pleiotropy. One outlier was identified for gastroesophageal reflux in the UK Biobank. The associations persisted after the removal of the outlier (Supplementary Table S13). With a strict linkage disequilibrium threshold, genetically predicted MVPA was still associated with these 8 identified gastrointestinal diseases (Supplementary Table S10). After adjustment for genetically proxied LST, although estimates were not substantially altered, the associations of genetically predicted MVPA with gastric ulcer and chronic gastritis were no longer statistically significant (Supplementary Table S11). The MR Steiger test confirmed the directionality of identified associations (Supplementary Table S12). Leave-one-out analysis did not find that a single instrument strongly influenced the identified associations (Supplementary Fig. S1).

### Pleitropic effects of smoking and alcohol consumption

When adjusting for genetically proxied smoking initiation, genetically predicted longer LST was associated with 14 of 24 gastrointestinal diseases except for alcoholic liver disease and cholangitis even though the estimates were modestly attenuated (Supplementary Table S14). Similar results were obtained after adjustment for genetically proxied cigarettes smoked per day (Supplementary Table S14). With adjustment for genetically predicted alcohol consumption, genetically predicted longer LST was still associated with 14 identified gastrointestinal diseases (Supplementary Table S14). After adjusting for genetically proxied smoking initiation, the associations of genetic predisposition to MVPA with gastroesophageal reflux, irritable bowel syndrome, cholecystitis, cholelithiasis, and acute pancreatitis remained (Supplementary Table S14). The associations for six gastrointestinal diseases remained significant after adjustment for genetically proxied cigarette smoked per day except for that for chronic gastritis (Supplementary Table S14). Similar findings were observed after adjustment for genetically predicted alcohol consumption (Supplementary Table S14).

### Mediation analysis

The mediation of BMI, WHR, type 2 diabetes, and fasting insulin in the associations between LST and 16 identified gastrointestinal diseases are shown in Table 1 and Supplementary Table S15. The proportion of BMI-mediation effect ranged from 0.95% for acute pancreatitis to 44.80% for acute appendicitis (Table 1). Similar to the pattern observed with BMI, the mediation effect proportion of WHR varied from 3.75% for cholecystitis to 43.01% for non-alcoholic fatty liver disease. The mediation effect of type 2 diabetes and fasting insulin was less pronounced than that of BMI. The proportion of the mediation effect of type 2 diabetes ranged from 0.86% for acute pancreatitis to 7.73% for duodenal ulcer, and the proportion of mediation effect of fasting insulin ranged from 0.26% for cholelithiasis to 15.77% for alcohol liver disease (Table 1).

**Table 1:** Proportion of mediation effect in association between leisure screen time and 14 gastrointestinal diseases via each mediator.

Disease/Mediator	Body mass index	Waist to hip ratio	Type 2 diabetes	Fasting insulin
Gastroesophageal reflux	–	4.31%	–	2.12%
Gastric ulcer	–	–	–	–
Duodenal ulcer	9.05%	10.42%	7.73%	–
Chronic gastritis	–	–	–	5.18%
Irritable bowel disease	–	–	–	–
Diverticular disease	8.38%	14.21%	1.26%	–
Crohn disease	11.77%	–	–	–
Ulcerative colitis	3.44%	5.32%	–	–
Non-alcoholic fatty liver disease	29.10%	43.01%	2.37%	–
Alcoholic liver disease	2.45%	–	–	15.77%
Cholangitis	32.57%	9.64%	3.31%	10.26%
Cholecystitis	25.34%	3.75%	–	–
Cholelithiasis	37.83%	16.14%	–	0.26%
Acute pancreatitis	0.95%	24.24%	0.86%	–
Chronic pancreatitis	28.64%	21.17%	–	–
Acute appendicitis	44.80%	6.84%	2.33%	9.92%

Missing values indicate that the total effect and the mediated effect are not aligned in the same direction, implying the absence of a mediating effect.

The results of the mediation analysis of BMI, WHR, type 2 diabetes, and fasting insulin in the association between MVPA and gastrointestinal diseases are shown in Table 2 and Supplementary Table S13. BMI and WHR separately mediated around 61.18% and 61.27% of the effect of MVPA on cholelithiasis (Table 2). The mediation effect of type 2 diabetes ranged from 5.20% for chronic gastritis to 49.82% for cholecystitis. Fasting insulin mediated the 6.79%–38.63% effect of MVPA on 8 gastrointestinal diseases (Table 2).

**Table 2:** Proportion of mediation effect in association between moderate-to-vigorous intensity physical activity and 8 gastroinestinal disease via each mediator.

Disease/Mediator	Body mass index	Waist to hip ratio	Type 2 diabetes	Fasting insulin
Gastroesophageal reflux	10.82%	3.45%	–	10.01%
Gastric ulcer	35.75%	47.46%	7.09%	38.63%
Chronic gastritis	26.73%	14.33%	49.82%	18.67%
Irritable bowel syndrome	34.97%	37.87%	16.45%	15.12%
Cholecystitis	28.83%	28.01%	10.43%	10.25%
Cholelithiasis	61.18%	61.27%	33.51%	–
Acute pancreatitis	40.41%	67.66%	5.20%	12.54%
Chronic pancreatitis	48.57%	34.89%	–	6.79%

Missing values indicate that the total effect and the mediated effect are not aligned in the same direction, implying the absence of a mediating effect.

## Discussion

This study comprehensively investigated the causal associations of LST and MVPA with the risk of 24 gastrointestinal diseases using MR analysis (Supplementary Fig. S2). Genetically proxied longer LST was associated with an increased risk of 16 gastrointestinal diseases. Genetic liability to MVPA was inversely associated with 8 gastrointestinal diseases. The associations for LST remained overall stable after adjustment for genetic liability to MVPA and vice versa. However, some associations attenuated after adjustment for genetically proxied smoking behaviors, which indicates the pleiotropic effects of smoking in this analysis. Multivariable MR analysis also found that genetically proxied BMI, fasting insulin levels, and type 2 diabetes partially mediated the associations of LST and MVPA with several gastrointestinal diseases.

The current MR found evidence that genetically predicted LST and MVPA were associated with several upper and lower gastrointestinal diseases. Previous observational studies suggested that high levels of physical activity were associated with a low risk of gastroesophageal disease,^2^^,^^28^ gastric ulcer,^29^ duodenal ulcer,^30^ diverticular disease^31^^,^^32^ and that lack of habitual physical exercise was associated with a higher risk of gastroesophageal reflux.^28^ The association between genetically predicted prolonged TV watching time and gastroesophageal disease was observed in previous MR analyses,^33^^,^^34^ which was consistent with our finding. Our MR study supported these findings and further corroborated the causality of these associations. We also provided evidence that genetically predicted LST was positively associated with some hepatobiliary and pancreatic outcomes including non–alcoholic fatty liver disease, alcoholic liver disease, cholecystitis, cholelithiasis, pancreatitis, and acute appendicitis. Genetically predicted MVPA was inversely associated with cholecystitis, cholelithiasis, and acute pancreatitis. A prospective cohort study including 360,047 participants showed that longer sedentary time was associated with a higher risk of chronic liver disease and replacing 1 h/day sedentary time with equivalent physical activity was associated with a lower risk of chronic liver disease,^31^ which was in line with our findings. Based on findings from the Nurses’ Health Study, sedentary behavior was independently associated with an increased risk of cholecystectomy.^35^ Extending the previous observational findings, our MR investigation strengthened the evidence that physical activity was associated with a low risk of cholecystitis^5^ and cholelithiasis.^6^ Our MR investigation also confirmed results from previous MR studies where genetically predicted longer television watching time was associated with higher levels of hepatic fat.^36^ Another MR study demonstrated that genetically predicted vigorous physical activity was associated with a decreased risk of non-alcoholic fatty liver disease.^37^ Our findings also provide novel evidence on the associations of LST and MVPA with acute appendicitis which need to be verified. Promoting physical activity and reducing sedentary behavior emerge as pivotal strategies in the prevention of a wide array of upper and lower gastrointestinal diseases, as well as hepatobiliary and pancreatic outcomes. Physicians should advise patients on the importance of regular physical activity and emphasize its role in reducing the risk of specific gastrointestinal diseases. Concurrently, public health initiatives may play a crucial role in gastrointestinal disease prevention by heightening awareness regarding the adverse consequences of prolonged sitting and by advocating the incorporation of physical activity into daily routines. These efforts collectively empower individuals to take proactive steps toward better gastrointestinal and overall health.

We did not observe positive associations of genetically predicted LST or MVPA with colorectal cancer or esophageal cancer, which was identified in previous observational studies.^38^^,^^39^ Besides, a previous MR analysis comprising 31,197 colorectal cases found that genetically predicted MVPA was associated with a decreased risk of colorectal cancer.^40^ This discrepancy is likely caused by a relatively small sample size of these cancers in our MR analysis.

Our results also provided information that genetically predicted longer LST was positively associated with the risk of several gastrointestinal diseases independent of MVPA and vice versa. These findings suggest that reducing sedentary time and increasing physical activity were both important for gastrointestinal disease prevention. Besides, after mutually adjusting for LST and MVPA, the association for chronic gastritis became null which could be explained by the similar constructs picked by LST and MVPA. Thus, the insignificant associations after mutual adjustment did not necessarily mean that either LST or MVPA was not a risk factor for chronic gastritis. In MVMR adjusting for smoking initiation and cigarettes smoked per day, some identified associations attenuated which indicated the potential horizontal pleiotropy introduced by smoking behaviors. Of note, we observed that BMI, fasting insulin, and type 2 diabetes seemed to partly mediate the associations of LST and MVPA with gastrointestinal diseases, which indicates that metabolic factors may be involved in the pathological process. In addition, given that a comparative proportion of the effect was mediated by type 2 diabetes, our results support the current guidelines that recommend increasing activity and decreasing sedentary behavior among type 2 diabetes patients from the American Diabetes Association to improve their overall well-being.^41^ Similarly, more physical exercise and less sedentary time should also be recommended among obese individuals as a part of the management of gastrointestinal health due to the mediation effects of BMI observed in the link from physical activity and sedentary lifestyle to gastrointestinal disease in this study.

Mechanisms linking sedentary behavior and physical activity with gastrointestinal disease are not completely understood. Apart from being overweight, animal experiments have suggested that prolonged sitting might disturb lipid metabolism and result in the reduction of lipoprotein lipase activity which then raises the possibility of impaired metabolic actions.^42^^,^^43^ Besides, there was evidence that sedentary behavior might lead to increased systemic chronic inflammation, which may also increase the risk of developing gastrointestinal diseases.^44^ In addition, high levels of physical activity and a shorter sedentary time have been associated with lower insulin levels,^45^ which have been indicated to play a role in the development of many gastrointestinal diseases.^46^, ^47^, ^48^ The diversity, composition, and functionality of gut microbiota can be modified by physical exercise possibly via intestinal barrier preservation and bile acid homeostasis improvement.^49^ In addition, standing up benefits physiological processes including postural blood flow, energy expenditure, and muscle contraction, which may promote glucose regulation, mitochondrial function, and endothelial function.^50^ A previous study found that independent of moderate-to-vigorous intensity activity time, increased breaks in sedentary time were beneficially associated with waist circumference, BMI, triglycerides, and 2 h plasma glucose.^51^ This may explain why adjustment for MVPA barely affected the positive association between LST and several gastrointestinal diseases.

Compared to physical activity, sedentary behaviors in relation to gastrointestinal diseases have been less investigated. Our findings on LST are potentially relevant to interventions and guidance aimed at reducing gastrointestinal diseases through behavioral intervention. Besides, LST has increased by approximately 1 h for young children and 0.7 h for adults during the COVID-19 pandemic,^52^ which may exacerbate these harms. According to our findings, emphasizing and adopting multilevel measures are critical to preventing unhealthy screen time.

The major strength of the present study is the MR design, which minimized bias from confounding and reverse causality and thus improved the causal inference in the associations of LST and MVPA with gastrointestinal diseases. We also used several independent outcome sources and combined the estimates, which increased statistical power as well as strengthened our findings by the observed consistency of results. Another strength is that we confined our analysis to individuals of European ancestry, which minimized the population stratification bias. Furthermore, the application of MVMR allowed us to probe into the mediation effects, uncovering potential mechanisms underlying these associations, thereby paving the way for targeted interventions and improved public health strategies. Meanwhile, conducting MVMR analysis conditionally on genetically correlated behavioral traits also minimized the pleiotropy effects, ensuring the robustness of our findings.

A major limitation of the current MR study was horizontal pleiotropy, which could affect the validity of our MR findings. However, we conducted a series of sensitivity analyses including adjustment for two smoking behaviors, and the consistent results indicated limited pleiotropy. Another limitation is the relatively limited sample size in several gastrointestinal diseases, which means this study might have had inadequate power to detect weak associations between the exposure and diseases. A relatively small number of instruments is available for MVPA which may result in inadequate statistical power and underestimating the impact of MVPA on gastrointestinal diseases. Besides, our study participants were of European ancestry, and thus the observed associations may not be directly generalizable to other populations. A further potential limitation is that individuals of the UK Biobank were included in both the exposure and outcome datasets, which might introduce sample overlap bias leading MR estimates towards observational estimates. However, this bias should be mitigated due to a good strength of the genetic instrumental variables used for the exposures (F statistic > 10).^53^ In addition, we detected consistent results using the outcome data without sample overlap in the exposure dataset (i.e., the FinnGen study). This sample overlap might lead to a winner’s curse and possible weak instrument bias in the multivariable MR analysis given some conditional *F*-statistics < 10. It is still noteworthy that the dichotomized measures of MVPA may misclassify intermediate levels of physical activity engagement and lose information on potential nonlinear exposure-risk effects or thresholds for gastrointestinal disease. Besides, if underlying biological relationships involve continuous gradations in activity levels and disease risk, it is also hard to interpret the causal effect estimate.^54^ Finally, the LST and MVPA were self-reported which could be biased by recall and awareness of the beneficial effects of physical activity.

### Conclusion

In conclusion, this MR investigation found that genetically proxied longer LST was associated with an increased risk of 16 gastrointestinal diseases, whereas genetic liability to MVPA is associated with a decreased risk of 8 gastrointestinal diseases. Considering that physical activity and sedentary behavior are inversely correlated, our findings support the independent benefits of reducing sedentary time and promoting physical acidity in gastrointestinal disease prevention. Future studies are encouraged to explore the joint effect of LST and MVPA on gastrointestinal health.

## Contributors

All authors read and approved the final manuscript and author contributions statement using CRediT with the degree of contribution:

Jie Chen (Conceptualization: Equal; Methodology: Equal; Formal analysis: Equal; Data curation: Equal; and Writing - review & editing: Equal).

Xixian Ruan (Conceptualization: Equal; Methodology: Equal; Formal analysis: Equal; Data curation: Equal; and Writing – original draft: Equal).

Tian Fu (Conceptualization: Equal; Methodology: Equal; Formal analysis: Equal; Data curation: Equal; and Writing – original draft: Equal).

Shiyuan Lu (Conceptualization: Supporting; Writing - review & editing: Supporting).

Dipender Gill (Conceptualization: Supporting; Methodology: Supporting; Writing - review & editing: Equal).

Zixuan He (Conceptualization: Supporting; Writing - review & editing: Supporting).

Stephen Burgess (Conceptualization: Supporting; Methodology: Supporting; Writing - review & editing: Equal).

Edward L Giovannucci (Conceptualization: Supporting; Methodology: Supporting; Writing - review & editing: Equal).

Susanna C. Larsson (Conceptualization: Equal; Methodology: Equal; Data curation: Equal; and Writing - review & editing: Leading).

Minzi Deng (Conceptualization: Equal; Data curation: Equal; and Funding acquisition: Equal; Writing - review & editing: Equal).

Shuai Yuan (Conceptualization: Leading; Data curation: Equal; Writing - review & editing: Leading).

Xue Li (Conceptualization: Equal; Data curation: Equal; Funding acquisition: Leading; and Writing - review & editing: Leading).

Jie Chen, Xixian Ruan and Tian Fu accessed and verified the underlying data.

## Data sharing statement

All data analyzed in this study can be obtained by a reasonable request to the corresponding authors.

## Declaration of interests

All authors declare no competing interests.
