# Supplementary figures and images for: Sedentary lifestyle, physical activity, and gastrointestinal diseases: evidence from mendelian randomization analysis
Source: eBioMedicine. 2024 Apr 6;103:105110. doi: 10.1016/j.ebiom.2024.105110 (PMC11004085; doi:10.1016/j.ebiom.2024.105110)

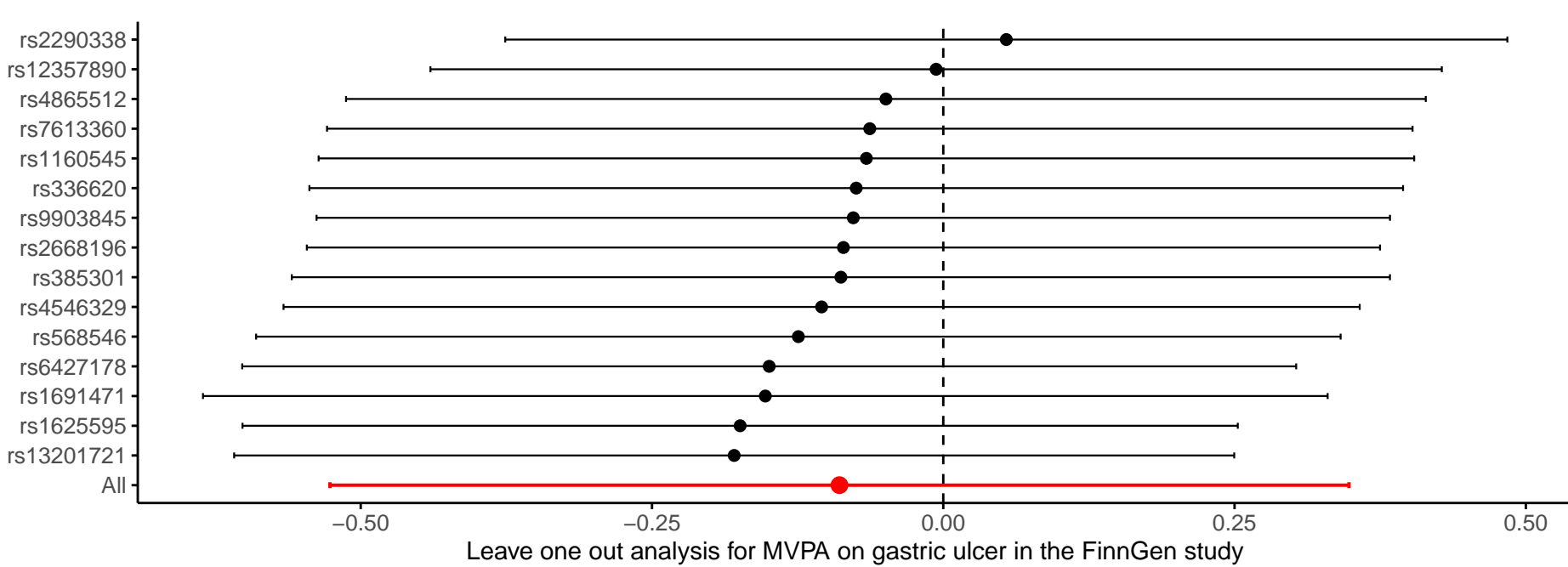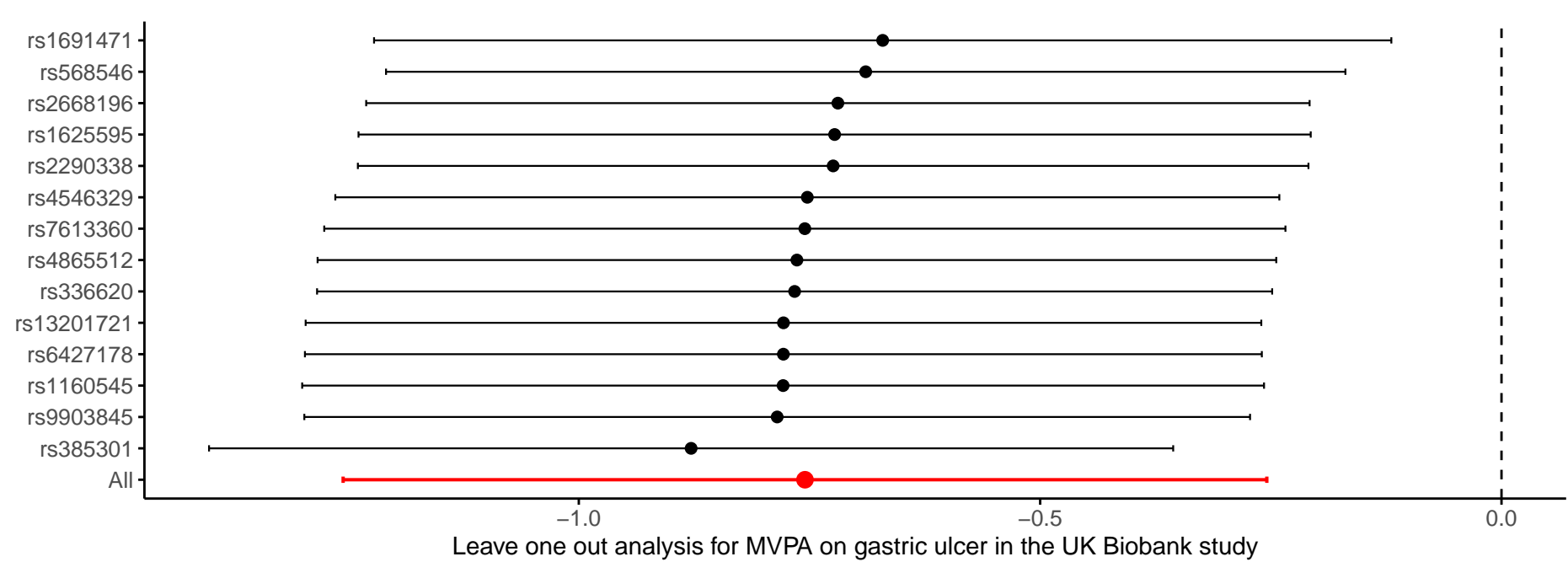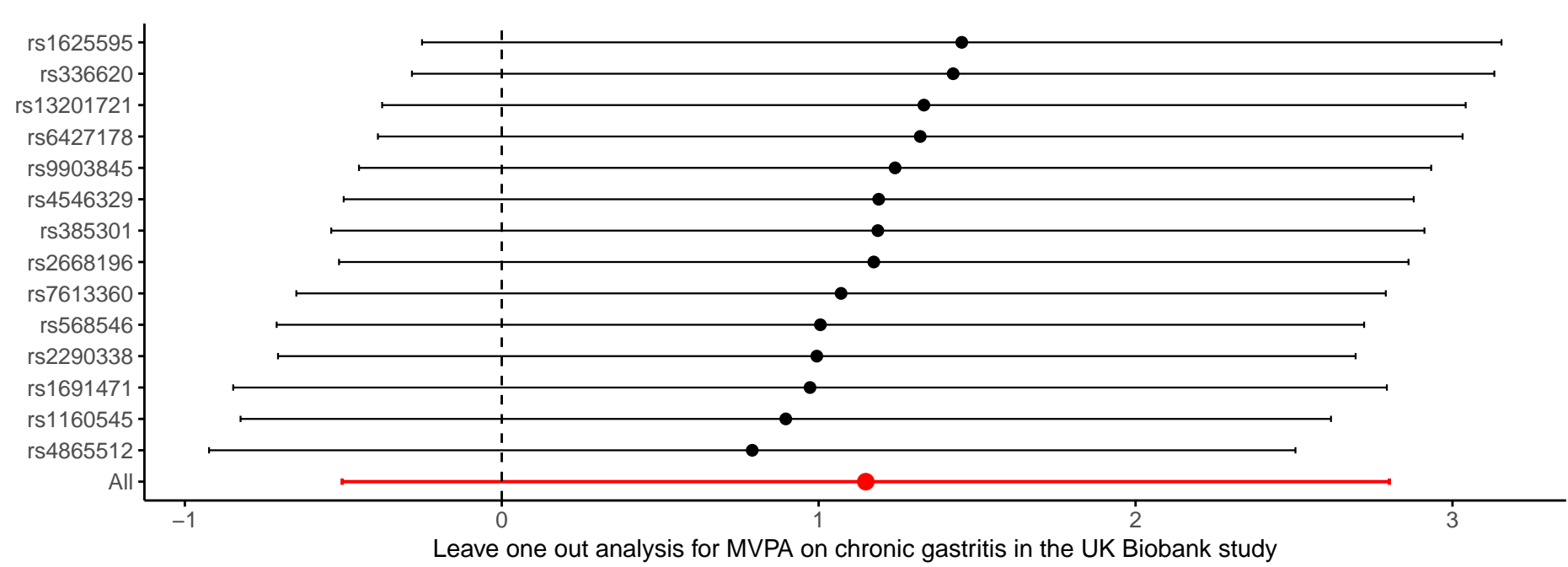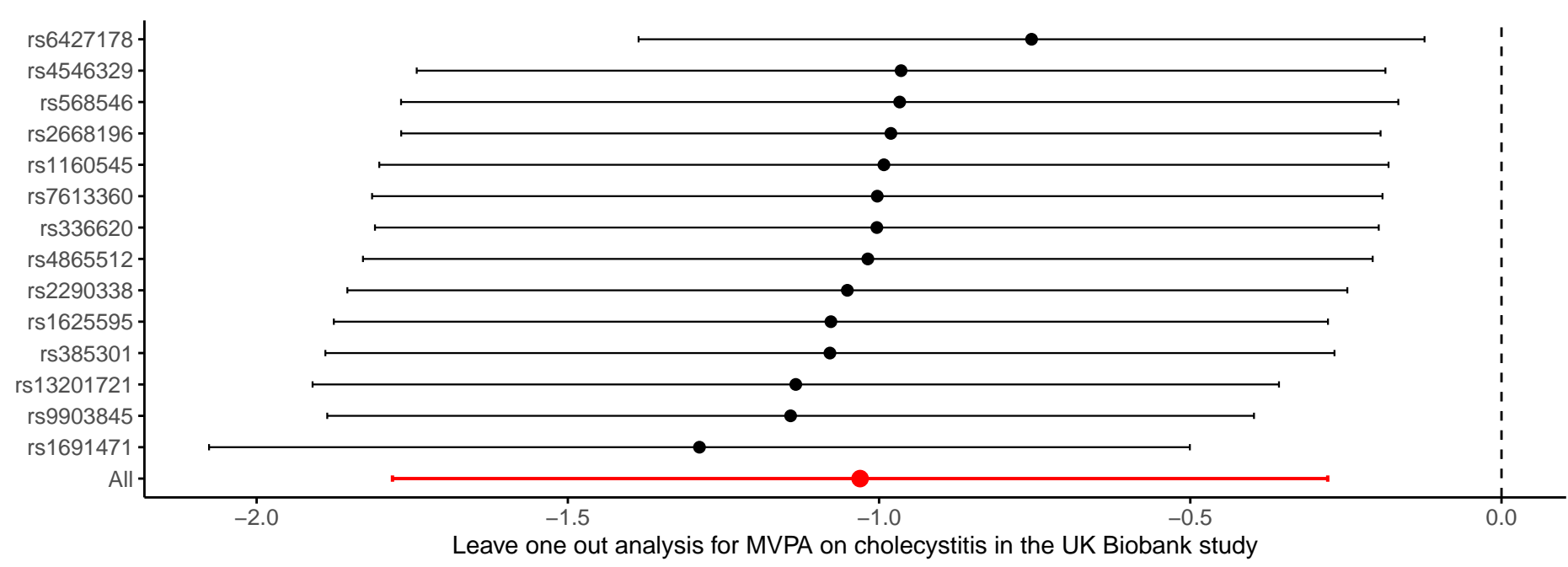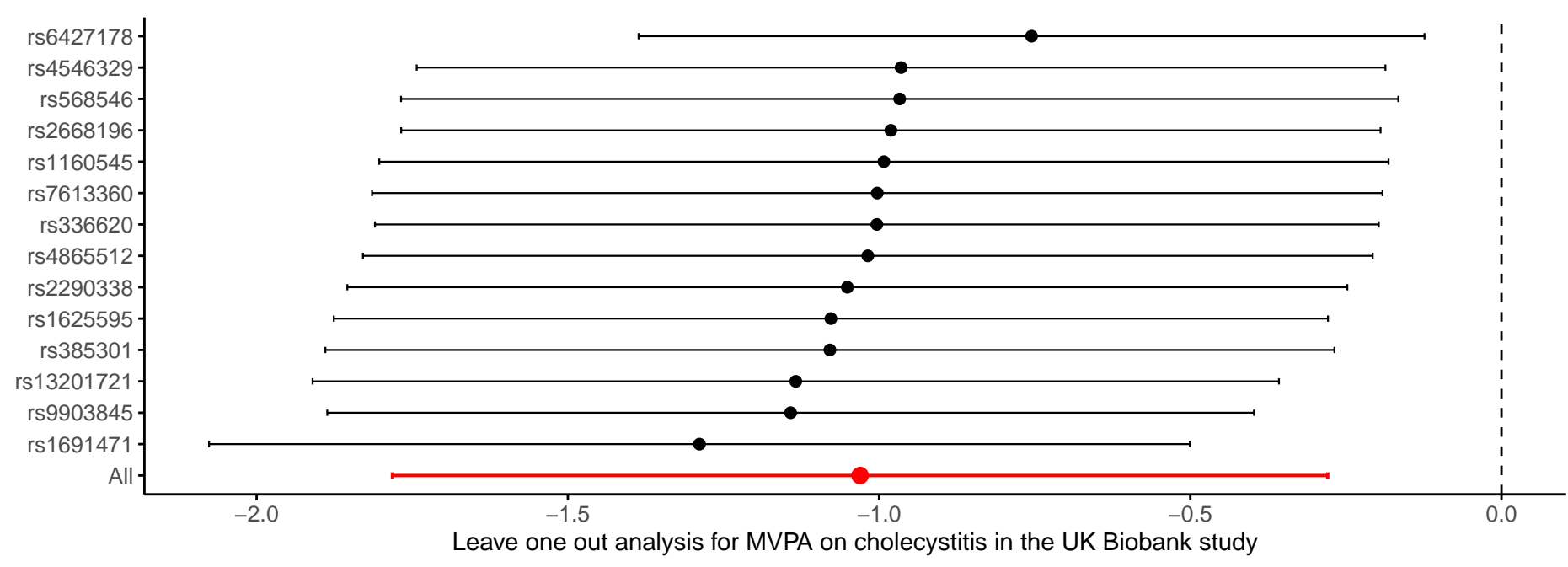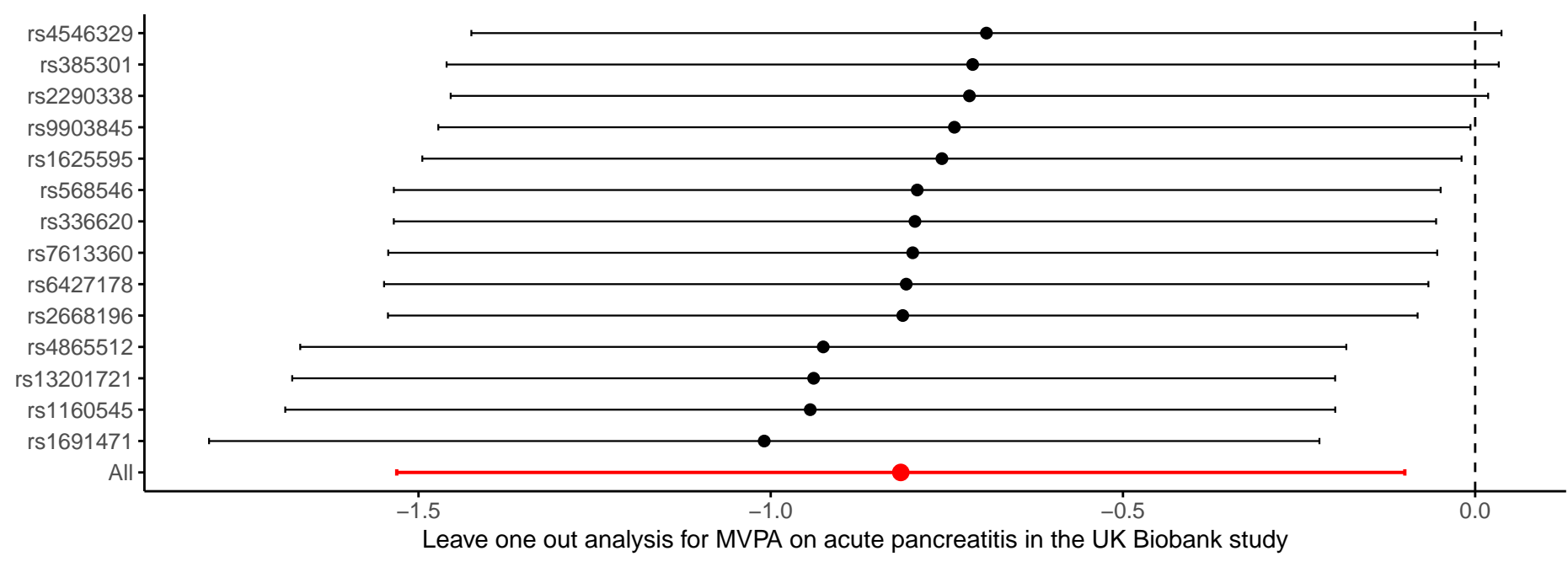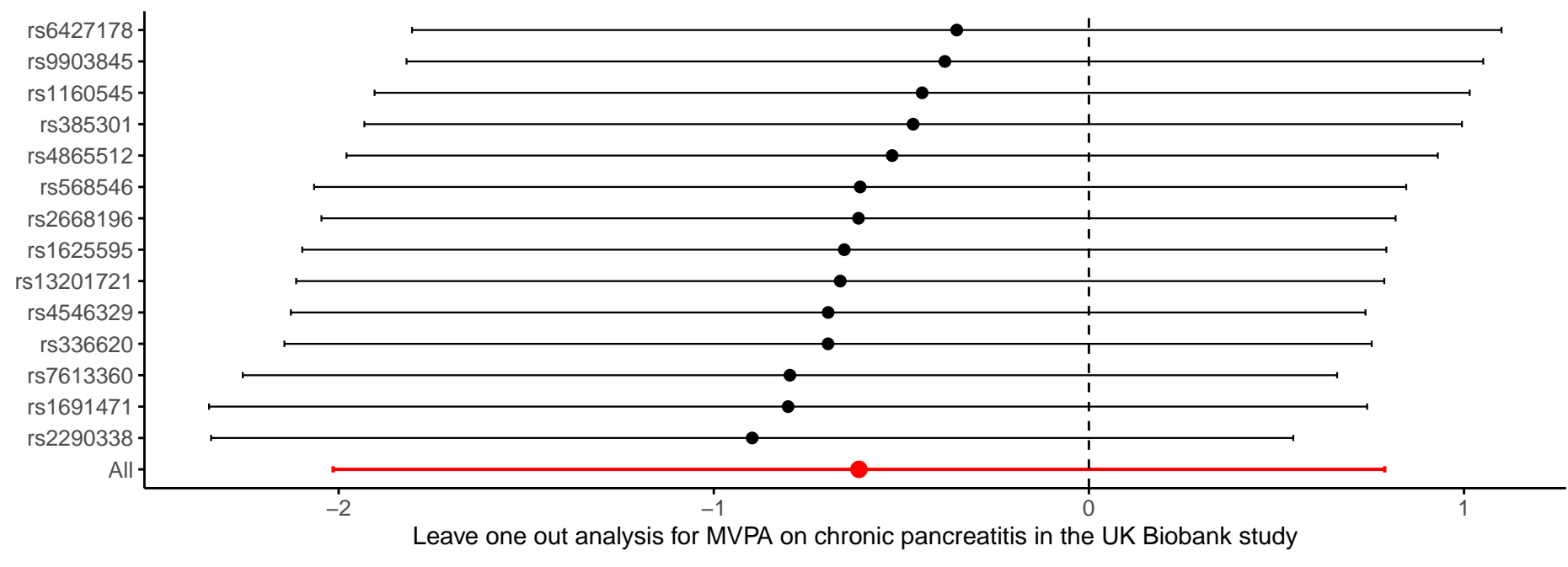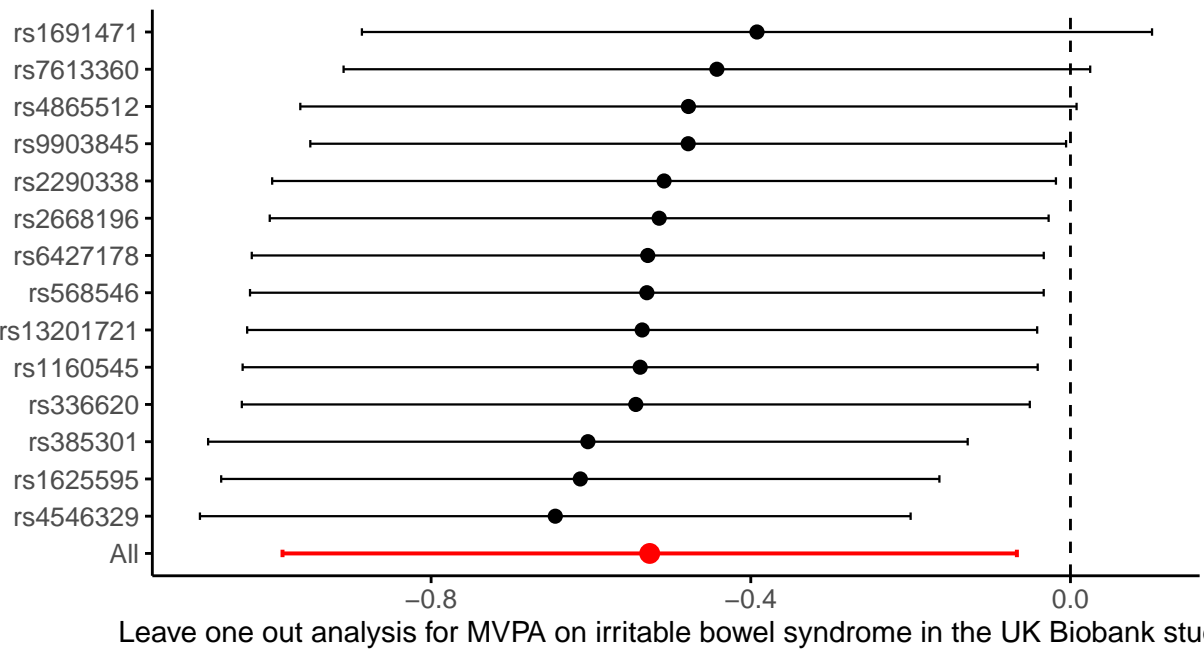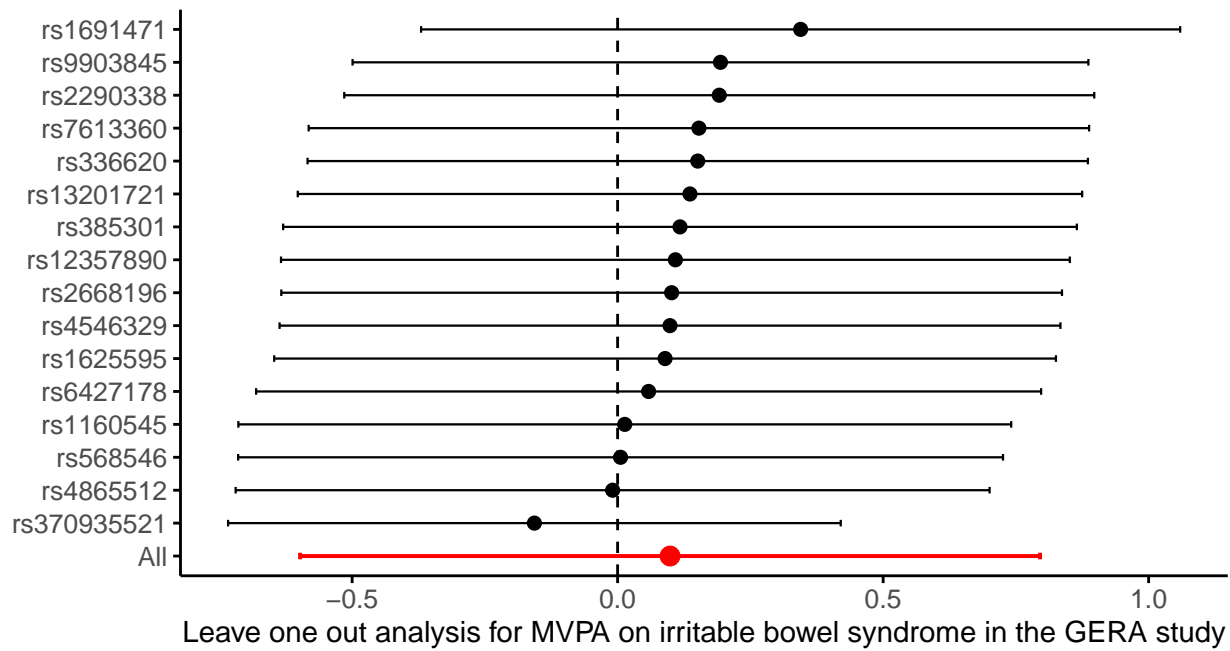

Supplement: Supplementary Fig. S1 [file mmc2.pdf]
