# Supplementary material for: Sedentary lifestyle, physical activity, and gastrointestinal diseases: evidence from mendelian randomization analysis
Source: eBioMedicine. 2024 Apr 6;103:105110. doi: 10.1016/j.ebiom.2024.105110 (PMC11004085; doi:10.1016/j.ebiom.2024.105110)
Supplement: Supplementary Fig. S2 [file mmc3.pdf]

- Significant positive association
- Suggestive positive association
- No significant association
- Suggestive inverse association
- Significant inverse association

	Main analysis	LD r^2=0.001	Adjustment for MVPA	Adjustment for smoking initiation	Adjustment for cigarette per day	Adjustment for drinking	Adjustment for BMI	Adjustment for WHR	Adjustment for type 2 diabetes	Adjustment for fasting insulin
Gastroesophageal reflux	1.28	1.30	1.16	1.25	1.26	1.27	1.30	1.27	1.31	1.27
Esophageal cancer	1.36	1.37	1.17	1.05	1.29	1.32	0.97	1.04	1.13	1.36
Gastric ulcer	1.26	1.20	1.19	1.20	1.25	1.26	1.35	1.26	1.28	1.27
Duodenal ulcer	1.41	1.46	1.42	1.39	1.33	1.39	1.37	1.36	1.38	1.44
Acute gastritis	1.15	1.15	1.01	1.05	1.13	1.08	1.04	0.98	1.06	1.12
Chronic gastritis	1.23	1.22	1.14	1.20	1.24	1.23	1.25	1.26	1.24	1.22
Gastric cancer	1.06	1.02	1.12	0.92	1.02	1.01	0.94	1.04	0.96	1.06
Irritable bowel syndrome	1.24	1.25	1.09	1.24	1.25	1.25	1.26	1.26	1.25	1.25
Celiac disease	0.96	0.95	0.88	0.94	0.95	0.94	0.81	0.74	0.85	0.96
Diverticular disease	1.26	1.27	1.21	1.23	1.25	1.25	1.24	1.22	1.26	1.28
Crohn's disease	1.18	1.11	1.09	1.22	1.21	1.27	1.16	1.22	1.25	1.23
Ulcerative colitis	1.18	1.13	1.06	1.14	1.11	1.15	1.17	1.17	1.16	1.13
Colorectal cancer	0.98	0.96	1.01	1.00	0.99	0.98	0.97	0.87	1.02	0.97
Non−alcoholic fatty liver disease	1.61	1.56	1.57	1.53	1.55	1.53	1.40	1.31	1.59	1.66
Alcoholic liver disease	1.24	1.27	1.15	1.03	1.24	1.23	1.23	1.34	1.30	1.20
Cirrhosis	1.18	1.18	1.20	1.11	1.20	1.17	1.16	1.23	1.22	1.17
Liver cancer	1.00	1.05	0.91	1.07	1.02	1.04	1.36	1.08	1.24	0.99
Cholangitis	1.36	1.28	1.28	1.22	1.34	1.36	1.23	1.32	1.35	1.32
Cholecystitis	1.31	1.26	1.16	1.23	1.28	1.27	1.23	1.30	1.34	1.33
Cholelithiasis	1.32	1.31	1.17	1.32	1.29	1.32	1.19	1.26	1.33	1.31
Acute pancreatitis	1.39	1.39	1.27	1.32	1.32	1.34	1.39	1.28	1.39	1.40
Chronic pancreatitis	1.47	1.39	1.37	1.34	1.40	1.41	1.32	1.36	1.49	1.50
Pancreatic cancer	1.09	1.09	1.05	1.03	1.06	1.12	1.03	1.09	1.12	1.12
Acute appendicitis	1.09	1.08	1.08	1.09	1.09	1.08	1.05	1.09	1.09	1.08

	Main analysis	LD r^2=0.001	Adjustment for LST	Adjustment for smoking initiation	Adjustment for cigarette per day	Adjustment for drinking	Adjustment for BMI	Adjustment for WHR	Adjustment for type 2 diabetes	Adjustment for fasting insulin
Gastroesophageal reflux	0.70	0.70	0.67	0.65	0.70	0.63	0.73	0.71	0.69	0.73
Esophageal cancer	0.77	0.69	0.50	0.61	0.70	0.78	0.75	0.80	0.83	0.79
Gastric ulcer	0.59	0.69	0.78	0.80	0.71	0.63	0.71	0.76	0.61	0.72
Duodenal ulcer	0.88	0.80	1.05	0.70	0.80	0.66	0.63	0.73	0.59	0.78
Acute gastritis	0.68	0.68	0.65	0.74	0.74	0.63	0.96	0.98	0.66	0.71
Chronic gastritis	0.74	0.73	0.74	0.81	0.85	0.73	0.80	0.77	0.86	0.78
Gastric cancer	1.27	1.31	1.26	0.92	1.47	1.09	1.47	1.51	1.31	1.43
Irritable bowel syndrome	0.60	0.62	0.62	0.64	0.53	0.62	0.72	0.73	0.65	0.65
Celiac disease	0.84	0.89	0.68	0.78	0.93	0.94	0.47	1.07	0.53	0.78
Diverticular disease	0.83	0.84	0.86	0.80	0.83	0.83	0.81	0.89	0.76	0.77
Crohn's disease	0.69	0.65	0.65	0.73	0.73	0.73	0.68	0.79	0.67	0.73
Ulcerative colitis	1.15	1.14	0.80	0.86	1.02	1.11	0.71	0.93	0.96	1.16
Colorectal cancer	1.05	1.06	1.08	1.05	1.09	0.99	0.86	1.07	0.94	1.06
Non−alcoholic fatty liver disease	0.68	0.70	0.93	0.62	0.58	0.60	0.71	0.70	0.80	0.84
Alcoholic liver disease	0.78	0.95	0.78	0.74	0.70	0.47	0.72	0.55	0.66	0.73
Cirrhosis	1.09	1.29	1.04	0.83	0.93	0.99	0.82	0.72	0.82	1.10
Liver cancer	0.54	0.45	0.60	0.41	0.35	0.56	0.70	0.63	0.52	0.91
Cholangitis	0.73	0.75	0.81	0.72	0.69	0.60	0.82	1.29	0.84	0.67
Cholecystitis	0.52	0.52	0.68	0.54	0.53	0.57	0.63	0.63	0.56	0.56
Cholelithiasis	0.61	0.61	0.62	0.70	0.66	0.65	0.83	0.83	0.72	0.61
Acute pancreatitis	0.52	0.54	0.66	0.71	0.56	0.64	0.68	0.81	0.54	0.56
Chronic pancreatitis	0.50	0.45	0.78	0.69	0.58	0.61	0.70	0.64	0.47	0.53
Pancreatic cancer	0.83	0.79	0.93	1.16	0.90	0.98	0.93	1.55	1.39	0.74
Acute appendicitis	0.83	0.84	0.95	0.78	0.81	0.87	0.87	0.86	0.87	0.89
